# Factors Influencing the Knowledge Gap regarding Influenza and Influenza Vaccination in the Context of COVID-19 Pandemic: A Cross-Sectional Survey in China

**DOI:** 10.3390/vaccines10060957

**Published:** 2022-06-16

**Authors:** Huimin Yin, Qingqing You, Jing Wu, Lianji Jin

**Affiliations:** 1 School of Journalism and Communication, Nanjing Normal University, Nanjing 210097, China; 200202034@njnu.edu.cn (H.Y.); 14190545@njnu.edu.cn (Q.Y.); 2 Faculty of Social Sciences, University of Ljubljana, 1000 Ljubljana, Slovenia; jw0822@student.uni-lj.si; 3 Law School, Yonsei University, Seoul 03722, Korea

**Keywords:** influenza, influenza vaccination, knowledge gap, vaccine hesitancy, health communication, COVID-19 pandemic

## Abstract

In the context of the COVID-19 global pandemic, promoting influenza knowledge and vaccine helps reduce the risk of dual pandemics and relieve the pressure on healthcare systems. Due to the low rate of influenza vaccination in China, we conducted a cross-sectional survey to investigate whether a knowledge gap regarding influenza and influenza vaccine exists between Chinese groups of different socioeconomic statuses and then explore the possible factors influencing knowledge level. A total of 951 valid questionnaires were collected online in this study. Variance analysis shows a knowledge gap regarding influenza and influenza vaccination between different socioeconomic status groups. Correlation analysis shows that internet media, social media, public communication, and interpersonal communication are positively associated with the knowledge level. Multilevel regression analysis shows a significant interaction between internet media and educational level. This study finds that internet media use helps narrow the knowledge gap between groups with different education levels. This article recommends a multi-channel promotion of influenza and vaccine knowledge and better pertinence between contents and readers.

## 1. Introduction

In the context of the COVID-19 global pandemic, influenza, as one of the major respiratory infections in the world [1], is estimated to cause 290,000 to 650,000 annual respiratory deaths worldwide [2], which cannot be ignored. On the one hand, influenza and COVID-19 have similar transmission routes, symptoms, and high-risk groups [3,4]. Influenza cases can affect the specificity and accuracy of COVID-19 surveillance [5]. On the other hand, if seasonal influenza and COVID-19 become a dual pandemic, it will place pressure on the current healthcare system [6]. Thus, it is necessary to focus on influenza prevention to reduce the potential harm of a dual pandemic.

Influenza vaccination is a cost-effective way of preventing influenza and reducing the risk of serious complications [2,7]. However, vaccine effectiveness largely depends on the vaccination rate [8]. Unlike the worldwide promotion strategy of the COVID-19 vaccine, the popularization of influenza knowledge and influenza vaccine has not received enough attention. In China, although some areas have launched free flu vaccination programs targeting certain groups such as the elderly, school-age children, and medical staff through livelihood projects, the influenza vaccination rate in China is still low, at only about 2%, on the basis of the 30.67 million doses supplied in 2019 [9].

There are various factors affecting vaccination rates, among which lack of knowledge about influenza and influenza vaccination is a critical reason [10]. Previous research on pregnant women [11] and healthcare workers [12] shows that the vaccination rate is positively associated with respondents’ knowledge scores about influenza and vaccines. To a certain extent, increasing influenza vaccination knowledge would change people’s risk awareness and improve their vaccination willingness [13]. Therefore, improving the public’s knowledge of influenza and influenza vaccines is significant in enhancing influenza vaccine coverage.

The traditional knowledge gap hypothesis points out that people with high socioeconomic status tend to acquire information faster than those with low socioeconomic status, whose ability to acquire knowledge is weak [14]. The knowledge gap between different groups has been documented, especially with respect to public services, scientific information, and health information [15]. Some scholars believe that the education level is the primary reason for the knowledge gap [16]. Education level may affect cognitive ability, media usage habits, previous knowledge, and social networks for information acquisition [17,18]. Then, the amount and type of media used to access the information also affect the formation and development of the knowledge gap [19]. For example, newspapers have been considered to widen the knowledge gap [20], while TV has been demonstrated to help bridge the knowledge gap [21,22]. Nowadays, new media such as the internet and social media are increasingly becoming important sources of health information [23], affecting the health knowledge gap. In addition to education and media usage, offline interpersonal and public communication has also been shown to affect the perception of influenza and influenza vaccination [24]. For example, reliable information from government, public health and medical experts can be cues for people to display healthy behaviors [25].

Improving the knowledge level of influenza and vaccine can help increase vaccination willingness [13], which is vital to influenza prevention, especially during the COVID-19 pandemic. Due to the limited number of related studies and the low influenza vaccination rate in China, this study takes the general population aged 18–65 in mainland China as the survey object to explore whether there is a knowledge gap regarding influenza and flu vaccination among different socioeconomic groups. If the knowledge gap exists, we will further explore the influencing factors, hoping to provide suggestions for future vaccine policymaking and publicity work.

## 2. Materials and Methods

### 2.1. Research Design

To measure respondents’ knowledge level about influenza and the flu vaccine, we designed and distributed a questionnaire through Wenjuanxing (https://www.wjx.cn/, accessed on 2 June 2022), one of the most well-known online survey platforms in China. The questionnaire consists of three parts: (1) Demographic profiles of respondents, including respondents’ gender, age, residence, occupation, monthly income, and education level. Income was compared using the purchasing power parity (PPP) (USD = 1) according to the International Comparison Program (ICP) 2017 cycle [26]. As the education level is often used to measure the socioeconomic status in previous studies about the knowledge gap [27], this study takes the same idea. (2) Evaluation of participants’ knowledge level. There are nine items in this section about influenza and six items about influenza vaccination (Table 1). Every respondent should judge whether each item is “right” or “wrong”.

The answers are compared with the correct answers, and correct answers score one point, while wrong answers and unanswered items score zero points, the total score measures the participant’s level of knowledge, and the full possible score is fifteen. (3) Respondents’ media usage. There are 18 options, divided into five sections: traditional media (radio, television, magazines, newspapers), internet media (search engines, portals, official websites of medical institutions, popular science websites), mobile social media (mobile clients, social media, online forums, mobile short videos), interpersonal communication (family, friends, professionals) and public communication (public places, grassroots organizations). The respondents receive one point for each choice and zero points for none. The total score can be used to evaluate a respondent’s degree of use of different media.

### 2.2. Sample

This study is a cross-sectional survey of people aged 18–65 in mainland China. From 1 January to 15 January 2022, we collected 951 valid questionnaires using the interpersonal snowball method through Wenjuanxing. Each participant read a consent statement before completing the questionnaire, which stated that participation was voluntary and that they could stop at any time. Clicking a “Next” button indicated that they agreed to continue completing the survey. The purpose of the study was also presented on the first page of the questionnaire. Because the subjects of this research are people aged 18–65 in the Chinese mainland, to make the age structure of the sample consistent with the population structure in the Chinese mainland, we reallocated the 951 questionnaires according to the results of “The Sixth National Population Census of China” [28]. Finally, 600 valid samples were kept (Table 2).

### 2.3. Data Processing

We checked abnormal values and multicollinearity recoded and centralized variables before the analysis. SPSS V.26 was used for descriptive statistics and variance analysis to determine whether there was an influenza and vaccination knowledge gap between groups with different education levels. Correlation analysis was used to determine the correlation between media use, interpersonal communication, public communication, and influenza knowledge score. Finally, multilevel regression analysis was conducted to determine whether different levels of media use, interpersonal communication, public communication, and other factors significantly predicted the level of influenza and vaccination knowledge. The variables were introduced into the multilevel regression model in the following order: Firstly, participants’ gender, monthly income, and education level were entered as control variables in Group 1. Secondly, traditional media, internet media, mobile social media, interpersonal communication, and public communication were entered into Group 2. Finally, education*interpersonal communication, gender*internet communication, gender*interpersonal communication, education*internet communication, income*internet communication, and income*interpersonal communication were entered as interactive variables in Group 3. The interactive variables were analyzed to test whether the above factors widened or narrowed the knowledge gap about influenza and vaccination between different education level groups.

## 3. Results

### 3.1. Reliability and Validity Test

This study assessed reliability and validity through confirmatory factor analysis (CFA) and Cronbach’s Alpha. As shown in Table 3, the Cronbach’s alpha value was between 0.71 and 0.81, which is higher than the threshold of 0.7, indicating that the overall reliability of the scale is high. Convergent validity was assessed by factor loading, AVE (Average Variance Extracted) and CR (Composite Reliability). The factor loadings exceeded 0.60 and were able to explain the structure well. In addition, the AVE values for each factor exceeded the benchmark value of 0.50, and the CR values exceeded the minimum benchmark [29]. The questionnaire had good convergent validity. In addition, the square roots of the AVEs of each factor were higher than the correlation coefficient of factors, indicating good discriminant validity. Therefore, the reliability and validity of this study passed the test.

### 3.2. Descriptive Statistical Analysis

The demographic characteristics of the 600 valid samples are shown in Table 4, of which 249 (41.5%) were males and 351 (58.5%) were females. After re-quota, the sample population matches the Chinese demographic structure. The largest population (29.0%) was aged 18–29 and the smallest population (7.0%) was aged 60–65. The regional distribution of the sample covered the administrative regions in mainland China; 16.7% of the respondents lived in Beijing, Shanghai, Guangzhou, and Shenzhen, and the proportion of the population living in provincial capitals and municipalities directly under the Central Government was 29.2%. After converting income into purchasing power parity (PPP) (USD = 1), the results show that 47.3% of the population had a monthly income of more than USD 1190.5. In comparison, 52.7% of the population had an income of fewer than USD 1190.5. The education level of the survey respondents covered five different levels, from junior high school and below to master’s degree and above. In addition, the undergraduate level had the highest coverage rate, reaching 43.2%, while those with junior high school education and below had the least, at 7.5%.

### 3.3. The Existence of the Knowledge Gap regarding Influenza and Influenza Vaccination

This study analyzed the education level and knowledge score by variance analysis to examine whether there was a knowledge gap. Results show significant differences in final knowledge scores among people with different education levels (Table 5). Respondents’ knowledge levels showed an increasing trend as their education levels increased, with respondents in junior high school and below having the lowest knowledge scores of influenza and flu vaccine (M = 2.72, SD = 1.02). Respondents with a bachelor’s degree (M = 3.77, SD = 0.95) and a master’s degree or higher (M = 3.76, SD = 0.89) had a higher knowledge level. This suggests a knowledge gap regarding influenza and influenza vaccination between groups with different education levels on the Chinese mainland.

### 3.4. Factors Associated with the Knowledge Level of Influenza and Influenza Vaccination

Internet media, mobile social media, public communication, and interpersonal communication were all positively correlated with the cognitive level of influenza and influenza vaccination. Based on this, this study then conducted a multilevel regression analysis of the independent and dependent variables. The correlation analysis results for the independent and dependent variables in this study are shown in Table 6.

The results of the multilevel regression analysis are shown in Table 7. In model one, the education level (β = 0.266, *p* < 0.01), monthly income (β = 0.158, *p* < 0.01), and gender (β = −0.093, *p* < 0.05) significantly predicted the knowledge score, explaining 12.8% of the outcome variable (R^2^ = 0.128). After controlling the second group’s education level, monthly income and gender, the explanatory power of independent variables for dependent variables increased by 5.7%, among which internet media (β = 0.148, *p* < 0.01) was able to significantly predict the knowledge score regarding influenza and influenza vaccination. In the third model, interactive variables were introduced, and the regression results showed a significant interaction between internet media and education level. The use of internet media was able to significantly predict the knowledge gap regarding influenza and influenza vaccination caused by education level.

**Figure 1:**
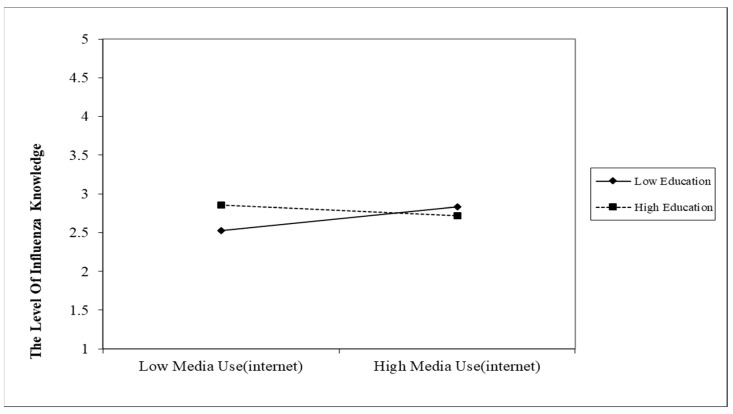
Regression plot for the interaction between media use (internet) and education.

Figure 1 further demonstrates the interaction between internet media usage and education level. Internet media use can help narrow the influenza and influenza vaccination knowledge gap. After reaching a critical point, there is even a reverse gap in knowledge scores between groups with high and low education levels. In other words, with the increasing use of internet media, the knowledge gap between people with different education levels becomes smaller. Even with the further use of internet media, the knowledge regarding influenza and influenza vaccination among the low-educated may exceed that of the high-educated group (β = −0.344, *p* < 0.05).

## 4. Discussion

### 4.1. Formation Mechanism of Knowledge Gap about Influenza and Flu Vaccination

Since the knowledge gap hypothesis was put forward, the health knowledge disparity has always been an essential part of knowledge gap research [30]. In this study of China, we find a knowledge gap regarding influenza and influenza vaccination. Respondents with junior high school education and below had the lowest influenza knowledge score (M = 2.72, SD = 1.02), respondents with undergraduate (M = 3.77, SD = 0.95) or master’s degree and above (M = 3.76, SD = 0.89) had a higher knowledge level. In other words, those with higher education levels have more knowledge about influenza and influenza vaccination than those with lower education levels. This further confirms the conclusion of previous studies that education level is an essential factor influencing the formation of a knowledge gap.

Unlike ordinary information, health information, such as vaccination-related information, usually contains complex scientific terms, and highly educated people tend to have good health science literacy and knowledge accumulation [18]. They are more likely to understand and accept expertise about influenza and vaccination than less educated people [31]. Additionally, people with higher education tend to have more information reference groups. A good level of social integration can give them more opportunities to discuss issues with knowledgeable people, thus going beyond the original social network and obtaining information support [18]. In addition to the existing knowledge and resource base, in some cases, the knowledge gap is caused by content preference. Previous studies have shown that highly educated groups are more likely to voluntarily access, receive and retain information about scientific content [30].

This study shows that the use of online media can significantly predict influenza and vaccination knowledge scores (β = 0.148, *p* < 0.01). In contrast, traditional media, interpersonal communication, and public communication cannot. This is consistent with the results of some previous studies, showing the importance of the internet in getting information [32]. With the increasing accessibility of network devices and the popularity of the internet, internet media has gradually become one of the essential sources for the public to access health information [33], which has profoundly influenced people’s knowledge and understanding of health issues [34]. Significantly, since the outbreak of COVID-19, home quarantine and other preventive measures have changed the way people learn and live. More and more people use the internet and social media to obtain health information. On the one hand, compared with traditional media, the content supplied by online media is vast and heterogeneous. Users can get much information independently and have deeper understandings [18], without relying on a single traditional media as in the past. On the other hand, compared with traditional media and offline channels, online media have functional advantages of immediacy, depth, and customization in information transmission [35], which are attractive. According to the “2020 National Health Search Data” released by Baidu Health, in the past five years, users’ search for health information such as medical checkups, medical consultations, and vaccines in Baidu has increased by nearly 200% [36]. Especially after the outbreak of COVID-19, the public demand for professional medical knowledge has been further strengthened, and the professional entry of the Baidu Health Medical Dictionary has exceeded 20 million daily page views [36]. In addition to general health information search, the internet also provides a platform for online consultation. The public can consult professional doctors online to obtain health information and accumulate relevant knowledge [37]. Given the significant influence of the internet on information acquisition, it is important to consider subjective factors such as reading skills, motivation, and media use when studying the formation mechanism and influencing factors of the knowledge gap.

### 4.2. Internet Media Usage Helps Narrow the Knowledge Gap

Internet media is an effective channel for information interaction and knowledge popularization. As the internet has become a significant health information source for the general public, the presentations and mechanisms of the health knowledge gap in the internet era have gradually become a hot topic. This study shows that the use of internet media can narrow the influenza knowledge gap between high- and low-educated groups.

In June 2021, the number of internet users in China reached 1.011 billion, and the internet penetration rate was 71.6% [38]. With the increasing accessibility of internet media, low-educated groups would have more opportunities to obtain health information through the network. Therefore, although the internet use skills of the low-educated group are not as good as those of the high-educated group, theoretically, as long as individuals use the media to search for influenza and influenza vaccination, they would increase relevant knowledge to some extent [39]. The “Online News Survey” conducted by China’s internet survey community shows that, compared with highly educated netizens, netizens with low education show a higher proportion of time surfing the internet through mobile phones and spend more time. They have more opportunities to watch news videos on mobile phones, news apps, and chat tools [40]. The internet media integrates various audio-visual elements and has diversified means of communication, improving users’ browsing interests and attracting more low-SES groups to learn health knowledge, such as that regarding influenza and influenza vaccination. Frequent browsing of health networks is associated with increased knowledge [41]. Especially during the COVID-19 global pandemic, these groups are more likely to be exposed to knowledge about infectious diseases and prevention. Paying attention to online news may increase the chances of low-educated groups expanding their original health knowledge and further narrowing the health knowledge gap with high-educated groups.

Besides the low-educated groups actively searching for information about influenza and vaccination, the ceiling effect of high-educated groups in the information search process will also affect the knowledge gap [42]. This is because knowledge spreads rapidly in the manner of asymptotes among higher-social-status groups. Once a group with high SES is satisfied it has acquired some specific information, it usually will not continue to learn; the acquisition of information will tend to the upper limit [43]. In most cases, the amounts and sources of information tend to increase only quantitatively, while the range, diversity, and thematic depth of arguments are usually limited. This means that even well-informed people cannot increase their knowledge further after reaching a certain level. In contrast, low-SES groups experience slower information diffusion, but their knowledge level is still likely to increase gradually [43]. While knowledge and information are not static, and gaps may continue to widen in other areas of expertise, in the case of influenza and vaccination, the knowledge gap between high- and low-SES groups is likely to narrow over time. In addition, although the groups with higher economic status can acquire information more quickly, misinformation and rumors in cyberspace may also increase their misunderstanding of influenza and influenza vaccine [44], impairing their knowledge level.

### 4.3. Suggestions for Bridging the Knowledge Gap regarding Influenza and Vaccination

Influenza is a respiratory infectious disease caused by the influenza virus, which is harmful to human health and to which all populations are susceptible [2]. The incidence of influenza in China increased yearly from 2015 to 2019, with approximately 253.4 influenza virus infections per 100,000 people in 2019. Although the incidence of influenza decreased in 2020 [45], this was partly due to preventive measures following the COVID-19 outbreak, such as home quarantine, wearing masks, etc. With the global risk of parallel transmission of COVID-19 and seasonal influenza, it is important to increase the influenza vaccination rate among the public, especially among high-risk groups, to create herd immunity and reduce the burden on the healthcare system. Therefore, it is important to bridge the knowledge gap regarding influenza and influenza vaccine between different socioeconomic status groups.

#### 4.3.1. Improve the Pertinence of Content According to the Target Audience

Since the education level affects the public’s acquisition and understanding of knowledge, groups with different educational backgrounds have different preferences for the content and form. Therefore, to achieve effective dissemination of information, media should be user-centered, and content editing should consider most users’ comprehension levels and preferences. At the same time, the media should combine its content style with the platforms’ characteristics. By improving the pertinence of content, the effective dissemination of content can be realized.

Since the low-educated group possesses fewer reading skills than the high-educated group, they are more easily attracted to entertainment content. Therefore, health communication targeting this group could expand the proportion of visual elements and simplify the narrative. In addition, more interesting presentation formats such as video, animation, and cartoon can be adapted appropriately to attract low-educated groups. As for the high-educated, media can increase the professionalism and depth of the content and provide more details, such as interviews with experts and the latest research results at home and abroad, and so on.

#### 4.3.2. Stimulate Individual Information Search Motivation

Although socioeconomic status may affect the speed of information acquisition and understanding level, when individuals start to collect information actively driven by intrinsic motivation such as curiosity and internal needs, they can obtain a deeper understanding and integration of information [46], which can help narrow the knowledge gap.

Therefore, in the current internet era, it is necessary to stimulate individuals’ motivation to search for health information and improve the initiative of low-SES groups to obtain information. Specifically, the government, hospitals, and communities can publicize the susceptibility and symptoms of influenza through offline communication and activity in winter and spring every year, the period of a high incidence of influenza in China, and focus especially on the harm of influenza spreading to high-risk groups. At the same time, it is necessary to introduce the importance and benefits of the influenza vaccine to residents and give timely responses to people’s doubts about vaccination. Schools, communities, and other institutions can also organize influenza knowledge competitions to attract more people to learn knowledge. The media can design interesting H5 games about influenza and vaccination. Encouragement from the external environment can stimulate internal motivation for learning knowledge, especially the interesting content that can attract the low-SES group. Combining online communication with offline activities can expand the reach of health information. It has specific significance for bridging the knowledge gap regarding influenza and influenza vaccination among different groups.

#### 4.3.3. Improve the Media and Health Literacy of Netizens

This study shows that internet media use can narrow the influenza knowledge gap caused by education disparity, which indicates the need to improve internet users’ media literacy and health literacy in the future. On the one hand, although the internet penetration rate in China is on the rise, the overall media literacy of Chinese netizens still lags behind that of developed countries due to the late start of media literacy education in China [47]. From the perspective of education level, China’s current internet users are mainly groups with secondary education levels. Netizens with junior high school, senior high school, technical secondary school, and technical school education account for 37.9% and 25.4%, respectively [48]. Improving netizens’ scientific literacy and media use skills is significant for bridging the health knowledge gap.

In the context of COVID-19, misinformation and rumors are spread widely on social media platforms. WHO defined this phenomenon as infodemic, which has become another enormous challenge in controlling the epidemic [49]. Similarly, misinformation and rumors about influenza and vaccination on the internet and social media may affect how people perceive health information and reduce their willingness to vaccinate against influenza. Therefore, while actively promoting the correct knowledge of influenza and the influenza vaccine, improving the public’s health literacy and detecting pseudoscientific information is also necessary. Specifically, the government can cooperate with the media to build authoritative popular science platforms to crack down on pseudoscience. Schools and communities can carry out offline health literacy education from time to time. Health organizations can set up public welfare internet health courses.

## 5. Conclusions

This study further proved the existence of a knowledge gap about influenza and influenza vaccination, which needs more attention during the COVID-19 pandemic. In this study, internet media, mobile social media, public communication, and interpersonal communication were positively correlated with influenza and influenza vaccination knowledge scores. There is a significant interaction between internet media and education level. Internet media can significantly predict the knowledge gap caused by education level. In addition, internet media use can help narrow the knowledge gap between groups with different education levels. These findings help us understand the knowledge gap regarding influenza and influenza vaccination and its formation mechanisms, providing suggestions for influenza vaccine promotion.

## Figures and Tables

**Table 1:** Questions about influenza and influenza vaccine.

No	Questions
1	Influenza is seasonal flu, an acute respiratory infection caused by the cold virus.
2	Unlike the common cold, high fever, headache, fatigue, cough, and general aches are the main symptoms of influenza.
3	Influenza usually heals itself in 3–14 days without causing severe illness or being life-threatening.
4	Influenza is highly contagious through droplet and contact transmission.
5	The whole population is susceptible to influenza.
6	Pregnant women, infants, the elderly, and people with chronic diseases are at high risk for severe illness and death from influenza.
7	Daily protective measures such as wearing masks, washing hands frequently, and keeping social distance can effectively reduce the infection and spread of influenza.
8	Annual influenza vaccination is the most effective measure to prevent influenza.
9	Drug prophylaxis can replace vaccination.
10	China has now approved a variety of influenza vaccines for the market.
11	Influenza vaccination is voluntary and self-funded in most regions of China.
12	Before the flu season every year, September and October are the best time to vaccinate against influenza.
13	Children aged from 6 months to 5 years are the key groups to receive the influenza vaccine.
14	The elderly aged 60 and above are the key groups for influenza vaccination.
15	Healthy adults should also be vaccinated against influenza.

**Table 2:** The age structure of the study participants (*N* = 600).

Age	Age Proportion of the Sixth Census (%)	Frequency (*N*)	Percentage (%)
18–29	20.32	174	29
30–39	16.14	138	23
40–49	17.28	144	24
50–59	12.02	102	17
60–65	5.08	42	7
Total	70.84	600	100

**Table 3:** Results of confirmatory factor analysis.

Construct	Item	Loading	Cronbach’s Alpha	Composite Reliability	AVE
Information Sources	IS	0.73	0.71	0.53	0.53
Knowledge	KL1	0.72	0.81	0.81	0.68
	KL2	0.92			

**Table 4:** Sociodemographic characteristics of the study participants (*N* = 600).

Variables	Category	Frequency (*N*)	Percent (%)
Gender	Male	249	41.5%
	Female	351	58.5%
Age	18–29	174	29.0%
	30–39	138	23.0%
	40–49	144	24.0%
	50–59	102	17.0%
	60–65	42	7.0%
Region	Beijing, Shanghai, Guangzhou, and Shenzhen	100	16.7%
	Provincial capitals and municipalities	175	29.2%
	prefecture-level city	161	26.8%
	County-level city	87	14.5%
	Town or village	69	11.5%
	other	8	1.3%
Monthly income (conversion to Purchasing power parity, US$ = 1)	<119.0	28	4.7%
	119.0–238.1	34	5.7%
	238.1–357.1	25	4.2%
	357.4–476.2	45	7.5%
	476.4–714.3	70	11.7%
	714.5–1190.5	114	19.0%
	1190.7–1904.8	120	20.0%
	1905.0–2381.0	50	8.3%
	2381.2–4762.0	94	15.7%
	>4762.0	20	3.3%
Education	Junior high school and below	45	7.5%
	High school, specialized secondary schools, skilled workers schools	86	14.3%
	Junior college	94	15.7%
	Undergraduate	259	43.2%
	Master’s degree and above	116	19.3%

**Table 5:** Results of variance analysis.

Educational Level	*N*	Mean	SD	Welch F	Sig
Junior high school and below	45	2.72	1.02	15.66	0
High school, specialized secondary schools, skilled workers schools	86	3.10	1.11		
Junior college	94	3.53	0.99		
Undergraduate	259	3.77	0.95		
Master’s degree and above	116	3.76	0.89		

**Table 6:** Correlation analysis between the independent and dependent variables.

Media	Knowledge				
Traditional media	0.042	**Traditional media**			
internet media	0.260 **	0.387 **	**Internet media**		
Mobile social media	0.236 **	0.268 **	0.513 **	**Mobile social media**	
public communication	0.191 **	0.285 **	0.417 **	0.397 **	**Public communication**
Interpersonal communication	0.201 **	0.185 **	0.325 **	0.366 **	0.408 **

** *p* < 0.01.

**Table 7:** Results of the multilevel regression analysis (*N* = 600).

	Model One					Model Two					Model Three				
	B	SE	t	*p*	β	B	SE	t	*p*	β	B	SE	t	*p*	β
(constant)	2.61 **	0.21	12.449	0		2.529 **	0.209	12.088	0		2.147 **	0.324	6.626	0	
Education	0.233 **	0.034	6.81	0	0.266	0.189 **	0.034	5.555	0	0.216	0.152 **	0.036	4.202	0	0.173
Monthly income	0.07 **	0.018	3.963	0	0.158	0.06 **	0.017	3.491	0.001	0.136	0.059 **	0.017	3.397	0.001	0.132
Gender	−0.193 *	0.081	−2.383	0.018	−0.093	−0.186 *	0.079	−2.345	0.019	−0.089	−0.182 *	0.079	−2.287	0.023	−0.087
Traditional media						−0.052	0.028	−1.897	0.058	−0.078	−0.055 *	0.027	−1.999	0.046	−0.082
internet media						0.101 **	0.032	3.153	0.002	0.148	0.248	0.149	1.663	0.097	0.365
Mobile social media						0.044	0.031	1.418	0.157	0.065	0.043	0.031	1.364	0.173	0.062
public communication						0.029	0.024	1.223	0.222	0.054	0.036	0.024	1.475	0.141	0.065
interpersonal communication						0.048	0.024	1.956	0.051	0.083	0.214	0.126	1.698	0.09	0.372
Education*Interpersonal											−0.031	0.021	−1.495	0.136	−0.205
Gender*internet											0.01	0.056	0.179	0.858	0.024
Gender*Interpersonal											0.005	0.047	0.115	0.908	0.015
Education*internet											−0.062 *	0.025	−2.447	0.015	−0.344
Income*internet											0.011	0.012	0.897	0.37	0.105
Income*Interpersonal											−0.009	0.01	−0.948	0.343	−0.109
R^2^	0.128					0.186					0.2				
Adjusted R^2^	0.124					0.174					0.181				
ΔR^2^	0.128					0.057					0.015				
F	F (3596) = 29.289, *p* = 0.000					F (8591) = 16.827, *p* = 0.000					F (14,585) = 10.458, *p* = 0.000				
ΔF	F (3596) = 29.289, *p* = 0.000					F (5591) = 8.277, *p* = 0.000					F (6585) = 1.787, *p* = 0.099				

Notes: Dependent variable: knowledge, * *p* < 0.05 ** *p* < 0.01.

## Data Availability

Data will be made available on special requests addressed to the corresponding author.
